# Identification of Serum Exosome-Derived circRNA-miRNA-TF-mRNA Regulatory Network in Postmenopausal Osteoporosis Using Bioinformatics Analysis and Validation in Peripheral Blood-Derived Mononuclear Cells

**DOI:** 10.3389/fendo.2022.899503

**Published:** 2022-06-09

**Authors:** Qianqian Dong, Ziqi Han, Limin Tian

**Affiliations:** ^1^ The First School of Clinical Medicine, Lanzhou University, Lanzhou, China; ^2^ Department of Endocrinology, Gansu Provincial Hospital, Lanzhou, China; ^3^ Clinical Research Center for Metabolic Disease, Gansu Provincial Hospital, Lanzhou, China

**Keywords:** postmenopausal osteoporosis (PMOP), circular RNA (circRNA), transcription factor (TF), competing endogenous network (ceRNA) network, bioinformatics analysis

## Abstract

**Background:**

Osteoporosis is one of the most common systemic metabolic bone diseases, especially in postmenopausal women. Circular RNA (circRNA) has been implicated in various human diseases. However, the potential role of circRNAs in postmenopausal osteoporosis (PMOP) remains largely unknown. The study aims to identify potential biomarkers and further understand the mechanism of PMOP by constructing a circRNA-associated ceRNA network.

**Methods:**

The PMOP-related datasets GSE161361, GSE64433, and GSE56116 were downloaded from the Gene Expression Omnibus (GEO) database and were used to obtain differentially expressed genes (DEGs). Gene ontology (GO) enrichment analysis and Kyoto Encyclopedia of Genes and Genomes (KEGG) pathway enrichment analysis were applied to determine possible relevant functions of differentially expressed messenger RNAs (mRNAs). The TRRUST database was used to predict differential transcription factor (TF)-mRNA regulatory pairs. Afterwards, combined CircBank and miRTarBase, circRNA-miRNA as well as miRNA-TF pairs were constructed. Then, a circRNA-miRNA-TF-mRNA network was established. Next, the correlation of mRNAs, TFs, and PMOP was verified by the Comparative Toxicogenomics Database. And expression levels of key genes, including circRNAs, miRNAs, TFs, and mRNAs in the ceRNA network were further validated by quantitative real-time PCR (qRT-PCR). Furthermore, to screen out signaling pathways related to key mRNAs of the ceRNA network, Gene Set Enrichment Analysis (GSEA) was performed.

**Results:**

A total of 1201 DE mRNAs, 44 DE miRNAs, and 1613 DE circRNAs associated with PMOP were obtained. GO function annotation showed DE mRNAs were mainly related to inflammatory responses. KEGG analysis revealed DE mRNAs were mainly enriched in osteoclast differentiation, rheumatoid arthritis, hematopoietic cell lineage, and cytokine-cytokine receptor interaction pathways. We first identified 26 TFs and their target mRNAs. Combining DE miRNAs, miRNA-TF/mRNA pairs were obtained. Combining DE circRNAs, we constructed the ceRNA network contained 6 circRNAs, 4 miRNAs, 4 TFs, and 12 mRNAs. The expression levels of most genes detected by qRT-PCR were generally consistent with the microarray results. Combined with the qRT-PCR validation results, we eventually identified the ceRNA network that contained 4 circRNAs, 3 miRNAs, 3 TFs, and 9 mRNAs. The GSEA revealed that 9 mRNAs participate in many important signaling pathways, such as “olfactory transduction”, “T cell receptor signaling pathway”, and “neuroactive ligand-receptor interaction”. These pathways have been reported to the occurrence and development of PMOP. To sum up, key mRNAs in the ceRNA network may participate in the development of osteoporosis by regulating related signal pathways.

**Conclusions:**

A circRNA-associated ceRNA network containing TFs was established for PMOP. The study may help further explore the molecular mechanisms and may serve as potential biomarkers or therapeutic targets for PMOP.

## Introduction

As one of the most common types of primary osteoporosis, postmenopausal osteoporosis (PMOP) is characterized by bone density reduction and bone microstructure deterioration, contributing to increased bone fragility and fracture risk (1, 2). PMOP-related fractures can affect individuals’ quality of life, increase the disability and mortality of patients, and cause high and rising economic and social burdens (3). PMOP is mainly caused by estrogen deficiency (4), which disrupts the homeostasis between bone formation facilitated by osteoblasts and bone resorption regulated by osteoclasts (5). However, the complex molecular biology mechanisms underlying PMOP have not been fully elucidated, and the potential therapeutic targets are limited. These factors hinder progress in the prevention and treatment of PMOP (6). Underlying molecular mechanisms need to be further explored and identify novel PMOP targets or biomarkers.

Notably, in recent years, various studies have indicated the promise of exosomes as affect therapies in osteoporosis (7). Exosomes are small, single-membrane organelles with diameters ranging from ∼40 to ∼160 nm (8). Exosomes carry diverse cargos, such as proteins, lipids, nucleic acids, and glycoconjugates (9). By releasing these substances, exosomes can remodel the extracellular matrix, and transmit signals or molecules between cells (10). In exosomes, circular RNAs (circRNAs) were recently identified as crucial cargos (11). CircRNAs are a vital class of non-coding RNAs, who are formed a close loop structure by reverse splicing of the 3′ end and 5′ end in the pre-mRNA (12). Highly conserved and widely expressed circRNAs can mediate protein translation, regulate gene transcription, and act as microRNA (miRNA) or protein sponges to inhibit their functions (13, 14). By modulating cell metabolism (15), cell proliferation (16), cell apoptosis (17), and other functions (18), circRNAs are involved in the incidence and development of some diseases, such as cancers (19), neurological diseases (20), heart diseases (21), and endocrine diseases (22), and circRNAs can function as therapeutic targets and biomarkers. Meanwhile, emerging researches indicated that circRNAs are also potential regulators of osteoporosis (23). For example, some studies revealed that hsa_Circ_0001275, hsa_circ_0002060, and hsa_circ_0006859 are recognized as potential novel diagnostic biomarkers of osteoporosis (2, 24, 25). CircFOXP1 can promote osteogenic differentiation of adipose-derived mesenchymal stem cells and bone regeneration by miR-33a-5p (26). Circ_0007059 can inhibit bone marrow stromal cells differentiation into osteoclasts by mRNA‐378/BMP‐2 axis (27). However, the functional roles of most circRNAs have not been clarified. Therefore, it is urgent and imperative to further explore the association between circRNAs potential functions and the mechanisms of osteoporosis.

Besides circRNA, microRNA (miRNA) is also one type of non-coding RNAs (ncRNAs) that contain about 22 nucleotides, who can repress stability and translation of downstream messenger RNAs (mRNAs) by binding to them (28, 29). Moreover, miRNAs also can regulate the expression of transcription factors (TFs), which in turn affect their corresponding target mRNAs (30). Some researches *in vivo* and *in vitro* have demonstrated that miRNAs are associated with the occurrence and development of osteoporosis (31–33). CircRNAs can act as competitive endogenous RNAs (ceRNAs) to competitive adsorb miRNA, thereby eliminating repressive effects of miRNA on its target mRNAs or TFs, regulating the expression of mRNA positively (34, 35). This mechanism is also known as the ceRNA hypothesis. So far, based on the ceRNA hypothesis, only a few studies have uncovered the functions of circRNA-related networks in osteoporosis (36, 37). And there is no report exploring the roles of circRNA-miRNA-TF-mRNA networks in osteoporosis. Therefore, systematic characterization of circRNA-miRNA-TF-mRNA regulatory networks may help further understand the molecular mechanisms and identify potential biomarkers of PMOP.

## Methods

### Data Acquisition and Processing

CircRNA microarray dataset (GSE161361), miRNA microarray dataset (GSE64433), and mRNA microarray dataset (GSE56116) were downloaded from Gene Expression Omnibus (GEO, http://www.ncbi.nlm.nih.gov/geo/). GSE161361 were obtained from GPL28148 Agilent-084217 CapitalBio Technology Human CircRNA Array v2, GSE64433 were obtained from GPL18402 Agilent-046064 Unrestricted Human miRNA V19.0 Microarray and GSE56616 were obtained from GPL11097 Custom Affymetrix Glyco v4 GeneChip.

GSE161361 contained serum samples of 3 PMOP patients and 3 matched healthy controls. GSE64433 contained 6 whole blood samples isolated from postmenopausal Chinese women with osteopenia or osteoporosis. GSE56116 contained peripheral blood samples from 10 patients with PMOP and 3 healthy postmenopausal controls.

The probe IDs of the original data were converted to official gene symbols by using data tables of microarray platforms with R software. Raw expression values were log 2 transformed with Aaffy package encoded by R. Finally, gene expression values were normalized using the normalize Between Arrays function of the R package limma.

### Identification of Differentially Expressed Genes

All samples were from serum and the samples were classified as PMOP and control groups (CON). The Limma package was utilized to screen DE circRNAs, DE miRNAs, and DE mRNAs between the PMOP group and CON group. The cutoff was set as |logfold change (FC)| > 2 and P value < 0.05 to identify DE circRNAs in GSE161361. To assess DE miRNAs in GSE64433, transcripts with a cut-off point of |logFC| > 0.5 and P value <0.05 were retrieved. In the analysis of DE mRNAs in GSE56616, |logFC| > 0.5 and P value <0.05 were implemented. Heatmaps and volcano maps of DE circRNAs and DE miRNAs were visualized using the “pheatmap” and “ggplot2” packages of R software, respectively.

### Functional Enrichment Analysis of DE mRNAs

Based on up-and downregulated DE mRNA transcripts, functional Gene Ontology (GO) enrichment analysis and Kyoto Encyclopedia of Genes and Genomes (KEGG) pathway enrichment analysis were performed, respectively. Enrichment analysis and drawing of bar graphs of GO and KEGG pathways were performed using the “org.Hs.eg.db” and “clusterProfiler” packages in R. GO terms and KEGG pathways were filtered at P < 0.05.

### Construction of TF-mRNA Network

The TF-mRNA relationship pair data were downloaded from the TRRUST database (https://www.grnpedia.org/trrust/). Findings of the RNA differential analysis were intersected with the TFs and corresponding mRNAs in the relationship pairs to obtain a differential TF-miRNA network. Mulberry mapping of differential TF-mRNA network was performed using the “ggalluvial” and “ggplot2” packages in R.

The miRNA-mRNA regulatory relationship pairs were downloaded from miRTarBase (https://mirtarbase.cuhk.edu.cn/). Then, target mRNAs of DE miRNAs were intersected with TFs and mRNAs of the TF-mRNA network that had been constructed in the previous step. Then, the miRNA-mRNA/TF regulatory relationship network was obtained.

### Construction of circRNA-miRNA-TF-mRNA Interaction Network

Information about circRNAs can be obtained in circBase (http://www.circbase.org/). For each DEcircRNA, all predicted target miRNAs were obtained by the circBank database (http://www.circbank.cn/). Next, miRNAs that overlapped with predicted and miRNAs in the miRNA-mRNA/TF network were gathered and the relative circRNAs in DE circRNAs were obtained. Finally, a ceRNA regulatory network in PMOP was constructed based on circRNA-miRNA pairs, miRNA-mRNA/TF pairs, and TF-mRNA pairs. The ceRNA network was visualized by Cytoscape version 3.8.0 software (https://cytoscape.org/).

### Verification of Selected mRNAs by CTD

Candidate mRNAs were further validated by Comparative Toxicogenomics Database (CTD) (http://ctdbase.org/) using the keyword “postmenopausal osteoporosis”. The inference score and reference count of candidate mRNAs were obtained.

### Patients and Samples

A total of 12 samples were collected in this study for candidate genes verification, including 6 PMOP patients and 6 healthy controls. Samples with other associated metabolic diseases, such as hyperparathyroidism, osteoarthritis, and diabetes were excluded. All the subjects underwent scanning of the total lumbar spine (L1–L4), total hip, femoral neck by dual X-ray absorptiometry (DXA). According to the WHO diagnostic classification, osteoporosis is defined by BMD at the hip or lumbar spine T-score≤ -2.5 SD. And the BMD diagnosis of normal is based on T-score of lumbar spine or hip at -1.0 SD and above. The detailed characteristics of the study subjects are summarized in **Supplementary Table 2**. The study was approved by Gansu Provincial Hospital.

### Verification of Candidate Genes by qRT-PCR

Peripheral blood was taken from each sample, and mononuclear cells were isolated. Total RNAs from mononuclear cells were extracted using TRIzol reagent (Takara, Japan), following the manufacturer’s protocol. 1 μg RNA from each sample was reverse-transcribed into cDNA using M5 Sprint qPCR RT kits with gDNA remover (Mei5 Biotech, Beijing, China) and miRNA 1st strand cDNA synthesis kits (Accurate Biology, Changsha, China). The qPCR reactions were performed with 1x Hieff qPCR SYBR Green Master Mix (Yeasen, Shanghai, China) on the ABI7500 Real-Time System (Applied Biosystems, American) according to standard protocols. Specific primers for mRNAs were synthesized by Sangon Biotech (Shanghai, China), and miRNA primers and circRNA primers were synthesized by Accurate Biology (Changsha, China). All specific primer sequences are listed in **Table 1**.

**Table 1:** Sequences of gene-specific primers used for qRT-PCR.

Gene	Sequence(5’→3’)	
	Forward	Reverse
**mRNA**		
β-actin ASNS CYP17A1 DAPK1 HGF HMOX1 HBB SLC19A1 PRL SPI1 TNFAIP6 TFPI2 VWF	TGGCACCCAGCACAATGAA GCTATGAAGATTGCACACAGAG CACCAACTATCAGTGACCGTAA GAGTTCTCTGGAAATCCTGTGT AATCCACTCATTCCTTGGGATT CCTCCCTGTACCACATCTATGT CACGTGGATCCTGAGAACTT CCAGCAAGAGCAGGTATGG CCACTACATCCATAACCTCTCC GCCCTATGACACGGATCTATAC GTTGCTTGGCTGATTATGTTGA TACAGTCCAAAAGATGAGGGAC CCTGTTACTATGACGGTGAGAT	CTAAGTCATAGTCCGCCTAGAAGCA ACAGAGCCACAAATACGGATAT TGATGATAACTTCTGTGCCCTT AAGACAACAACATGGATTGACG TCCCATTTACAACTCGCAATTG GCTCTTCTGGGAAGTAGACAG CCAGCCACCACTTTCTGATA CCACTGCATTCTCGGTTTTG GTTGATGGCCTTGGTAATGAAC AAGTCCCAGTAATGGTCGCTAT CTCATCTCCACAGTATCTTCCC GAATTTTCCGGATTCTACTGGC CATGAAGCCATCCTCACAGTAG
**TF**		
BACH1 CEBPB NFIC POU2F2	CTCTGAGTACTGAAGGCTGTTC CATCGACTTCAGCCCGTAC CTACCCACCTCATCTCAACC TGGACCAGACACTAATCATCAG	GAGTCGTCTCCCAAGCTAATG GAGAAGAGGTCGGAGAGGAAG GAGCTGACCACTTCCATTTAAC TCAGCCTTGATCTTTGTACTGG
**circRNA**		
hsa_circ_0023417 hsa_circ_0078309 hsa_circ_0063533 hsa_circ_0036760 hsa_circ_0086166 hsa_circ_0039035	ATCCATGGCACTGAAGAGGG CCAACCAGTGCACCATTGAT AGCTCAACAACTGTGGCATG GATCCAGGACATCGAGGGAG ATCAAAAAATTCACATGGGATAGAC TGCTTCCCTTGCTCTCTGAG	GCCAGTGGAAAGTAACCCCA GGCCGGCTTTCTCTAATGTG TTGGGACTGGCACTAACTGT TCCCCGGAAATCTGTTGGT ACGTGTTCTGGCCGAGAGAC CTCACTCCCTAGACCTGTGC
**miRNA**		
hsa-miR-4768-3p hsa-miR-629-3p hsa-miR-623 hsa-miR-566	CCAGGAGATCCAGAGAGAAT GTTCTCCCAACGTAAGCCCAGC ATCCCTTGCAGGGGCTGTT GGGCGCCTGTGATCCCAAC	

Relative transcript levels of circRNAs and mRNAs were normalized with β-actin, and U6 was employed as an internal control of miRNAs. The expression level of each mRNA, miRNA, and circRNA was calculated using the 2^−ΔΔCt^ method.

### Gene Set Enrichment Analysis

Gene set enrichment analysis (GSEA) was performed with the “clusterProfiler (v4.2.2)” package in R software. The annotated gene set (c2.cp.kegg.v7.5.1.entrez.gmt) was downloaded from the GSEA website (http://www.broadinstitute.org/gsea/index.jsp) as the reference gene set. “Ggplot2” and “enrichplot” R packages were applied to visualize the GSEA results. FDR (q value) < 0.25 and P < 0.05 were considered statistically significant.

## Results

**Figure 1:**
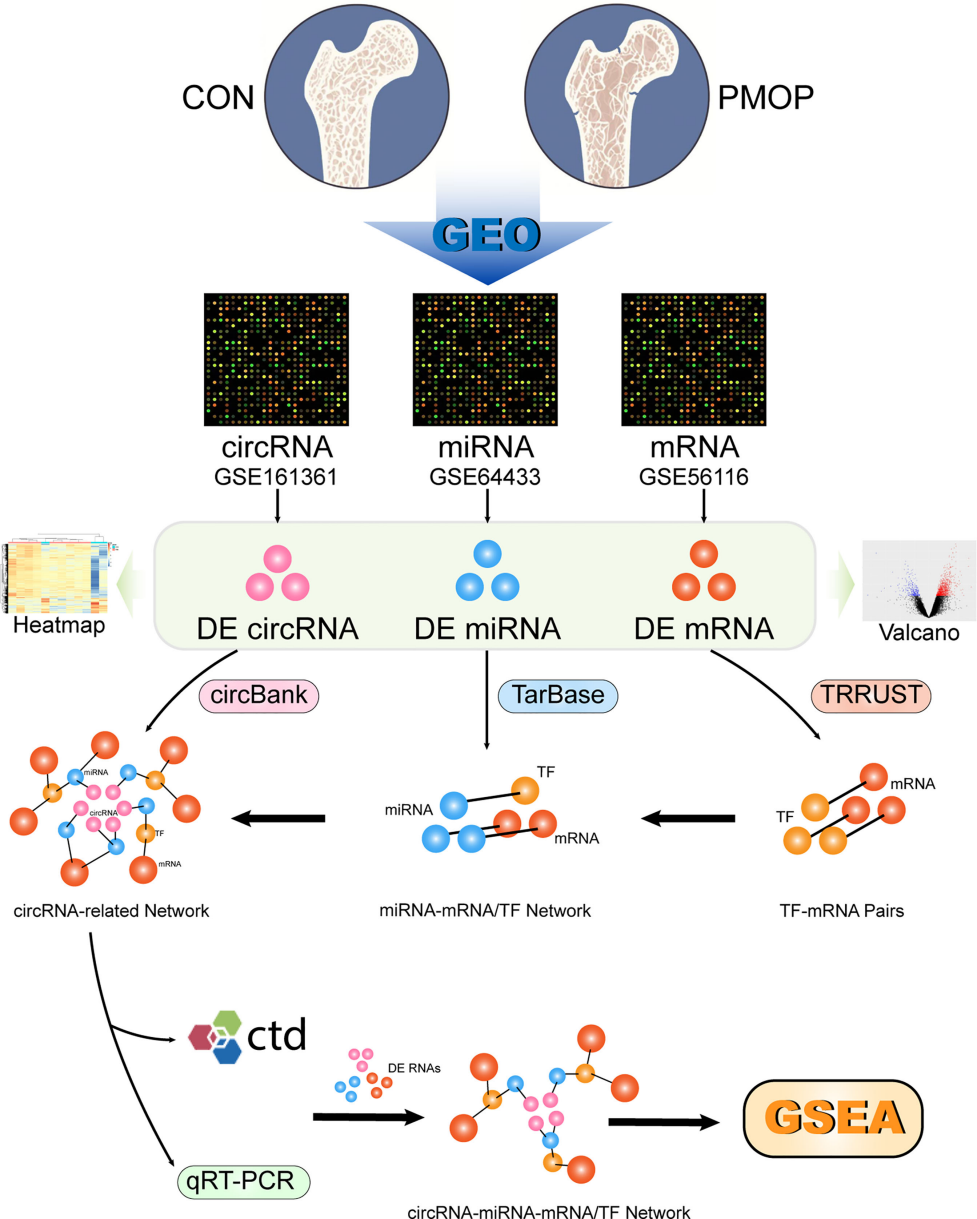
Schematic presentation of the analysis process.

The general scheme of the method used in this study is shown in **Figure 1**. The three datasets were used for differential analysis of mRNA, miRNA, and circRNA, respectively, and a circRNA-miRNA-TF-mRNA network was constructed based on the database interaction results.

### Data Download

We downloaded the gene expression profile of PMOP with the accession number GSE56116 from the GEO database, which contained 10 peripheral blood samples from patients with PMOP, and 3 healthy postmenopausal controls. Peripheral blood miRNA expression matrices of 3 PMOP patients and 3 matched controls were downloaded from the GSE64433 dataset for miRNA analysis. Serum samples of 3 PMOP patients and 3 matched healthy controls were downloaded from the GSE161361 dataset for exosomal circRNA analysis.

### Results of the DEGs Analyses

**Figure 2:**
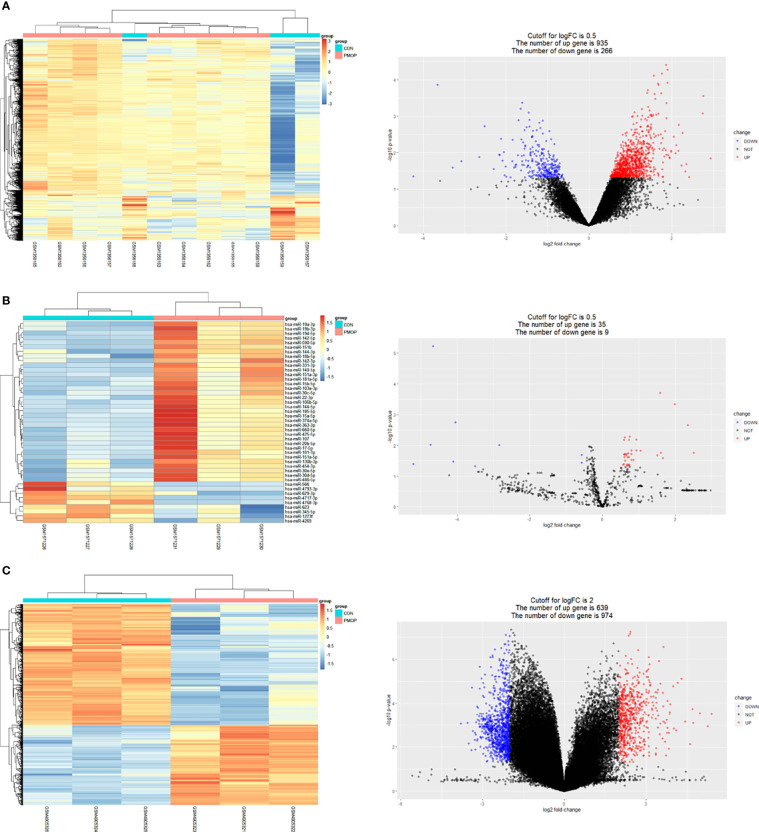
Results of differential analysis and volcano plot for mRNA, miRNA, and circRNA. **(A)** Heat map showing differential mRNA expression levels on left. Volcano plot showing fold differences in gene expression and P value relationship for the significance test on the right. **(B)** Heat map showing differential miRNA expression levels on left. Volcano plot showing fold differences in miRNAs expression and P value relationship for the significance test on the right. **(C)** Heat map showing differential circRNA expression levels on left. Volcano plot showing fold differences in circRNAs expression and P value relationship for the significance test on the right. Red represents upregulated expression, and blue represents downregulated gene expression in all volcano plots.

The results for all DEGs analyzed are shown in **Table 2**. Results of mRNA and miRNA differential analysis are shown in the heatmap and the volcano plot (**Figures 2A, B**). A total of 1201 DE mRNAs and 44 DE miRNAs were obtained by filtering at P < 0.05 and log2FC > 0.5, respectively. Results of circRNA differential analysis are shown in the heatmap and the volcano plot (**Figure 2C**), filtered by P < 0.05 and log2FC > 2 to obtain 1613 DE circRNAs.

**Table 2:** Statistical analysis of all differentially expressed ncRNAs and mRNAs.

Expression RNAs	Total No	No. upregulated	No. downregulated	Most upregulated (P value)	Most downregulated (P value)
circRNA	1613	639	974	hsa_circ_0047341 (0.000313768)	hsa_circ_0014219 (0.000902399)
miRNA	44	35	9	hsa-miR-18b-5p (0.017421388)	hsa-miR-4793-3p (0.040633291)
mRNA	1201	935	266	C17orf87 (0.014557551)	TMEM119 (0.044540982)

### Functional Enrichment Analysis

**Figure 3:**
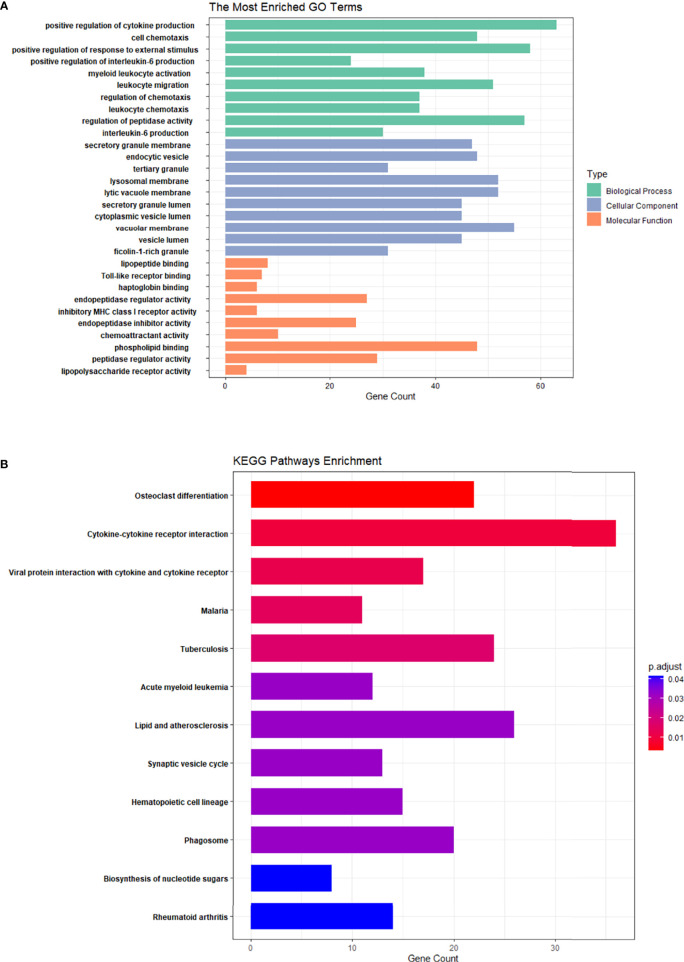
GO enrichment analysis and KEGG enrichment analysis of differential mRNA in PMOP and control groups. **(A)** The top 10 GO enrichment analyses of biological processes, cellular components, and molecular functions. **(B)** KEGG enrichment analysis of differential mRNA in the PMOP and control groups.

GO and KEGG enrichment analyses for DE mRNAs were performed. Bar graphs represent the top 10 most significantly enriched biological processes (BPs), cell components (CCs), and molecular functions (MFs) (**Figure 3A**). In the PMOP group, BPs of DE mRNAs determined by GO analysis were mainly “positive regulation of cytokine production”, “active regulation of IL-6 production”, “regulation of chemotaxis”, and “regulation of peptidase activity”.

The mainly enriched cell components (CCs) included “secretory granule”, “endocytic vesicle”, “tertiary granule”, and “lysosomal membrane”. The molecular functions (MFs) were mainly enriched “Toll-like receptor binding”, “endopeptidase regulator activity”, “endopeptidase inhibitor activity”, and “chemoattractant activity”.

KEGG pathway analysis (**Figure 3B**) revealed that the DE mRNAs were mainly enriched in pathways, involving “osteoclast differentiation”, “rheumatoid arthritis”, “hematopoietic cell lineage”, and “cytokine-cytokine receptor interaction”.

### Identification of TF-mRNA Regulatory Pairs

**Figure 4:**
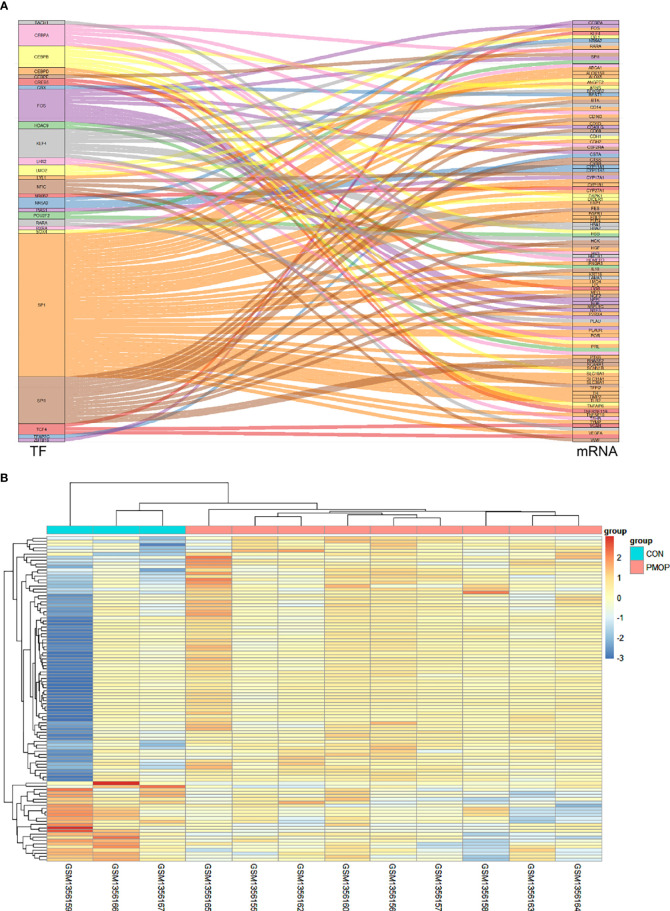
Construction of TF-mRNA network. **(A)** A Sankey diagram showing the predicted differential TF-mRNA regulatory relationships according to the TRRUST database. **(B)** The heatmap for the gene transcripts expression in the TF-mRNA regulatory network in the PMOP group and CON group.

A total of 118 TF-mRNA regulatory pairs were obtained after intersecting with differentially expressed mRNAs from the TRRUST database. A Sankey diagram shows the relationships between 26 TFs and their target mRNAs (**Figure 4A**). The heatmap shows the relative gene expression levels in the TF-mRNA regulatory network (**Figure 4B**).

### Construction of miRNA-mRNA/TF Network

**Figure 5:**
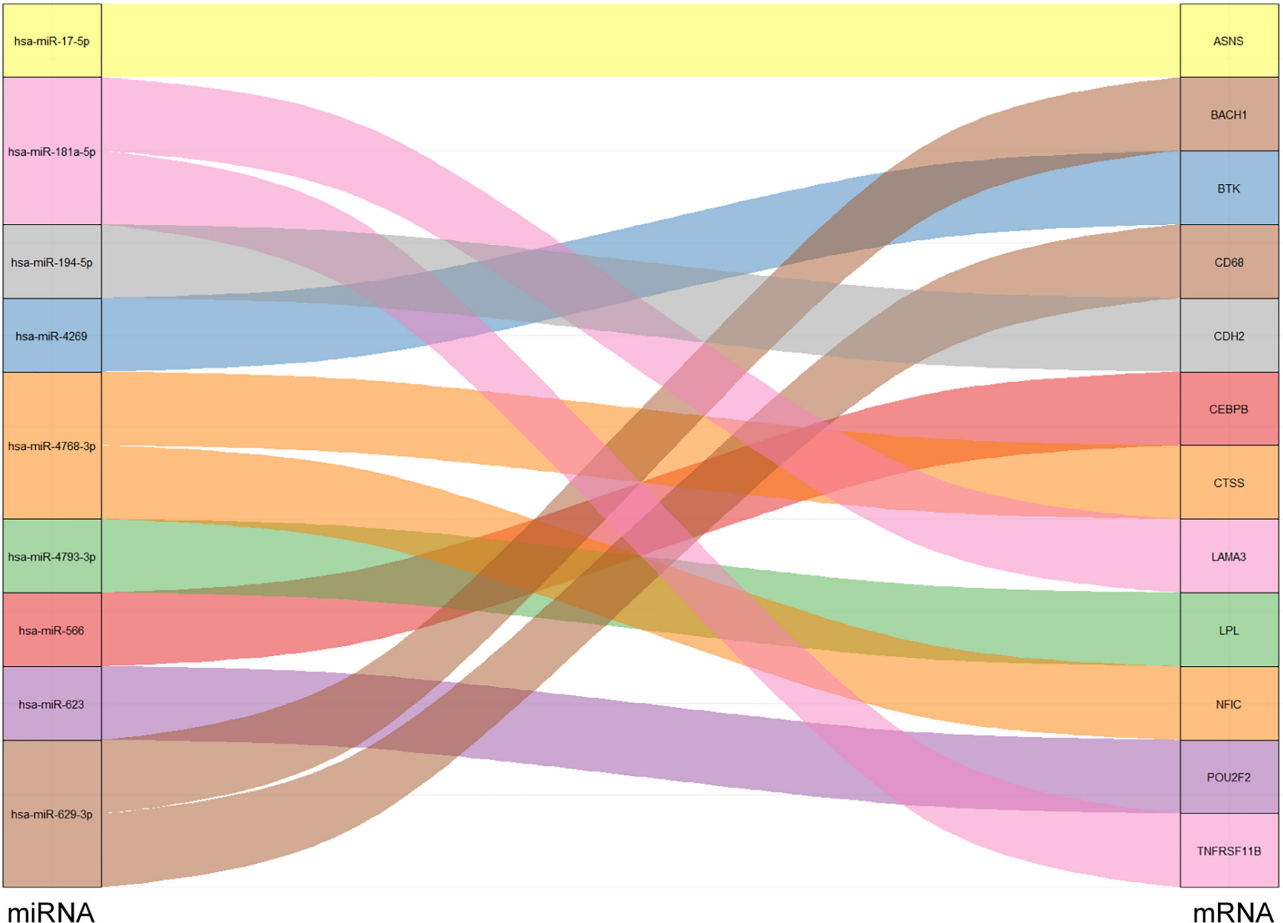
A Sankey diagram showing results of miRNA -TF/mRNA (containing TF) regulatory pairs.

Based on DE miRNAs, target genes were predicted using the miRTarBase databases. The predicted genes were intersected with the above differential TF-mRNA network to obtain 12 differential miRNAs and their corresponding target mRNAs (containing TF). A Sankey diagram shows the differential miRNA-mRNA/TF regulatory relationship pairs (**Figure 5**).

### Construction of the circRNA-Related Network

**Figure 6:**
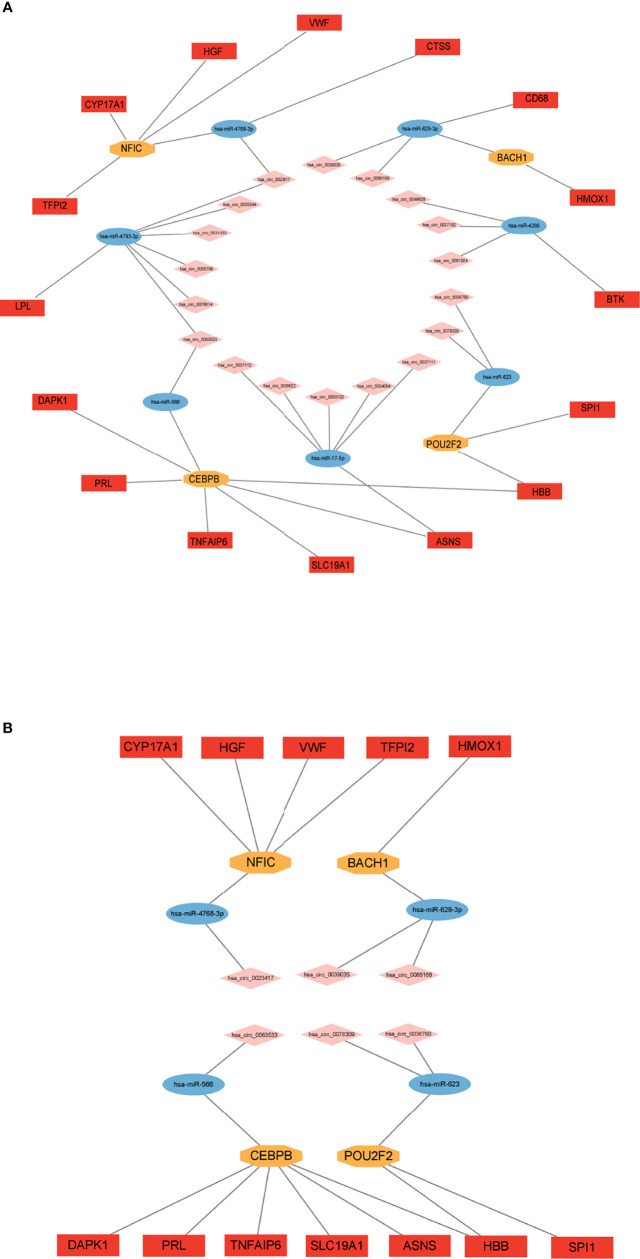
Construction of circRNA regulatory network. **(A)** circRNA-miRNA-TF/mRNA regulatory network. **(B)** circRNA-miRNA-TF-mRNA regulatory network.

With bioinformatics prediction from circBank, circRNAs that can target miRNAs of the above miRNA-mRNA/TF regulatory relationship were obtained. Combining DE circRNAs from dataset GSE161361, we identified 18 circRNAs. According to the above regulatory pairs, the Cytoscape software was used to map and construct the circRNA-related network (**Figure 6A**). Then regulatory relationship pairs that did not contain TFs were excluded. Finally, we built a circRNA-miRNA-TF-mRNA network, which contains 6 circRNAs, 4 miRNAs, 4 TFs, and 12 mRNAs (**Figure 6B**).

### CTD Analysis of Candidate mRNAs

By mining the CTD using the keywords “postmenopausal osteoporosis”, a total of 14,521 potential target genes of PMOP were obtained. 12 mRNAs and 4 TFs in the above circRNA-related network were also identified in the database. We found that these genes were associated with PMOP with different inference scores and reference counts (**Table 3**).

**Table 3:** The candidate genes associated with PMOP in CTD.

Gene Symbol	Inference Score	Reference Score
**mRNA**		
ASNS	12.16	16
CYP17A1	14.57	5
DAPK1	14.6	4
HGF	25.57	18
HMOX1	23.38	20
HBB	10.16	3
SLC19A1	20.17	17
PRL	23.96	18
SPI1	7.44	2
TNFAIP6	23.25	6
TFPI2	10.68	3
VWF	19.22	17
**TF**		
POU2F2	2.67	1
NFIC	2.52	1
BACH1	6.56	2
CEBPB	27.14	19

### qRT-PCR Verification of the Key Genes of the ceRNA Network

**Figure 7:**
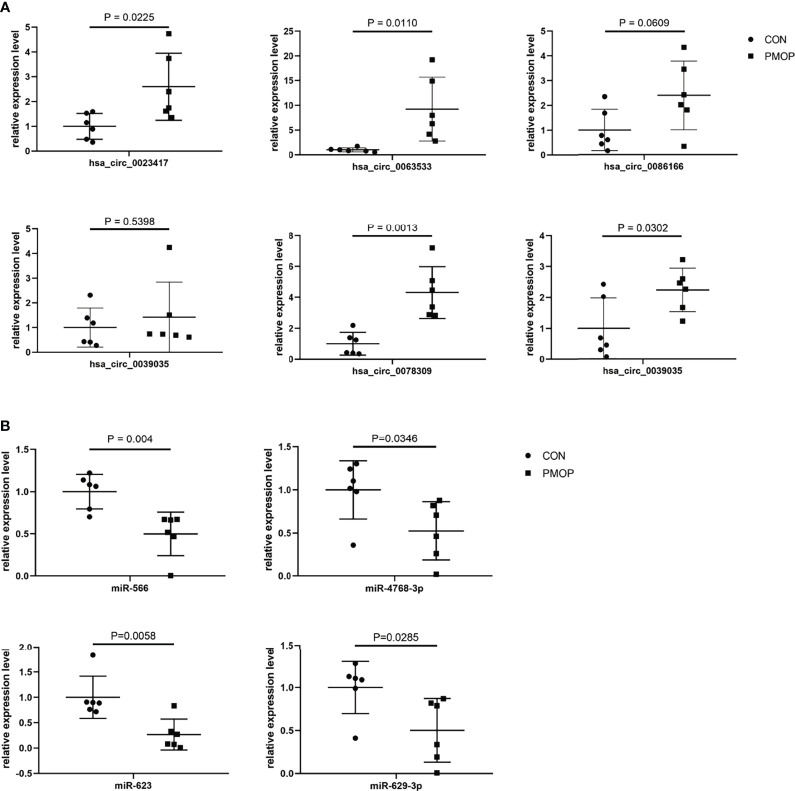
qRT-PCR experiment validation. **(A)** The expression levels of circRNAs. **(B)** The expression levels of miRNAs.

**Figure 8:**
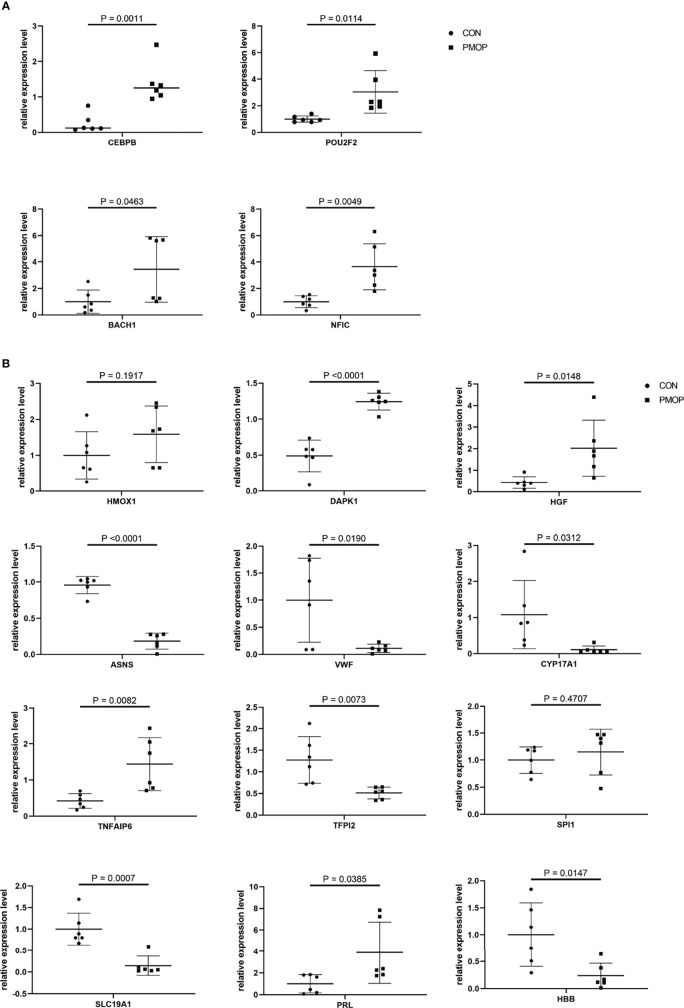
qRT-PCR experiment validation. **(A)** The expression levels of TFs. **(B)** The expression levels of mRNAs.

We verified the relative gene expression levels of the key genes in the ceRNA network by qRT-PCR. There was no statistically significant difference in the expression of hsa_circ_0039035 and hsa_circ_0086166 between the PMOP and the control group. Other circRNAs, such as hsa_circ_0023417, hsa_circ_0078309, hsa_circ_0063533, and hsa_circ_0036760 were up-regulated, which were consistent with microarray data (**Figure 7A**). The qPCR results showed the expression levels of hsa-miR-566, hsa-miR-623, hsa-miR-629-3p, and hsa-miR-4768-3p were generally consistent with the microarray results (**Figure 7B**). The qRT-PCR results also showed similar expression trends of TFs to microarray analysis (**Figure 8A**). Of the 12 mRNAs examined with qRT-PCR, 3 miRNAs did not match the results of the microarray: HMOX1, SPI1, and SLC19A1(**Figure 8B**).

### Construction of the circRNA-miRNA-TF-mRNA Network

**Figure 9:**
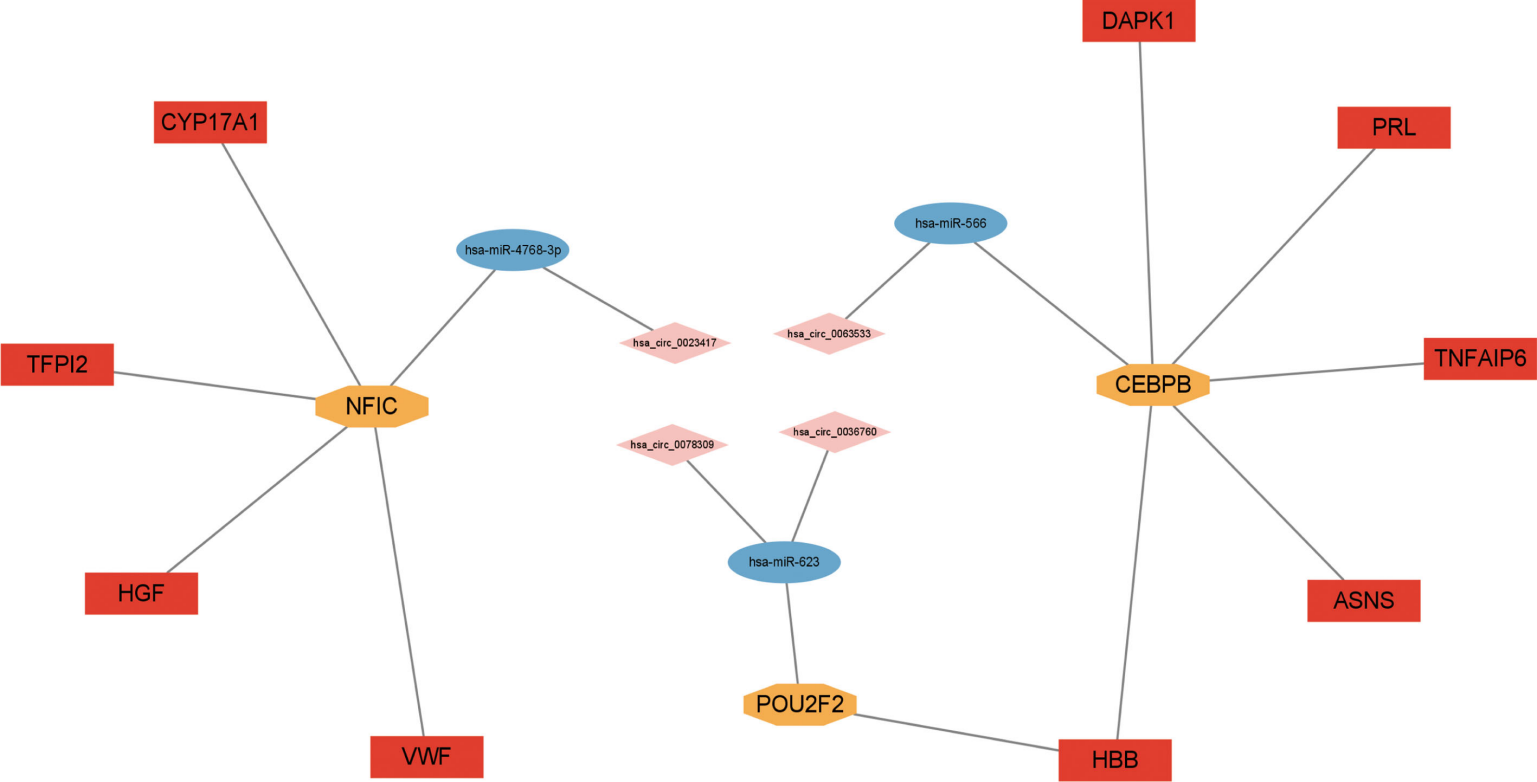
Construction of circRNA-miRNA-TF-mRNA regulatory network.

By combining the qRT-PCR validation results, the circRNAs and mRNAs that did not agree with the microarray analysis trend were excluded. Finally, a ceRNA network containing 4 circRNAs, 3 miRNAs, 3 TFs, and 9 mRNAs was obtained (**Figure 9**).

### Gene Set Enrichment Analysis

**Figure 10:**
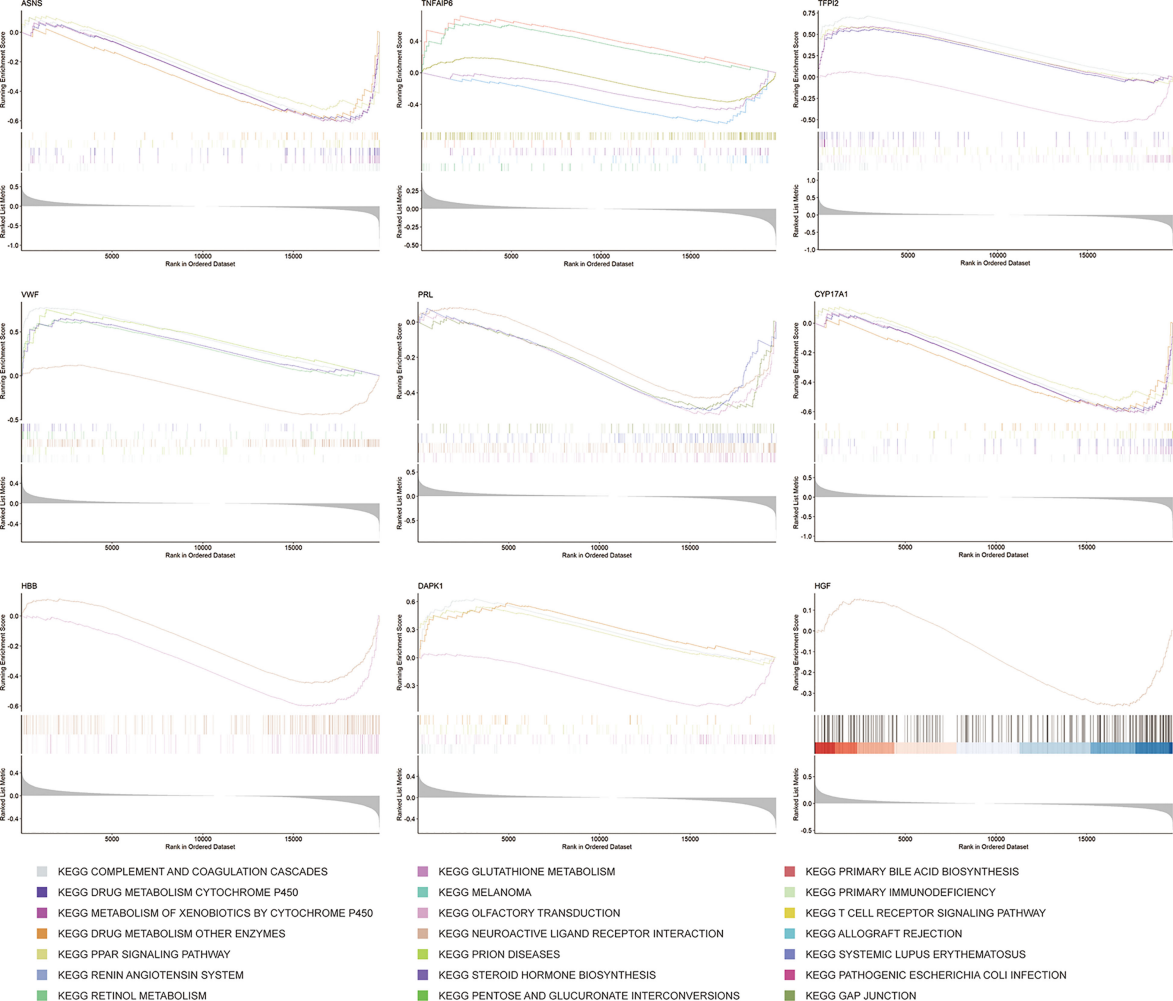
Barcode plots showing the presentative results of GSEA.

GSEA is a method that confirms whether a given set of genes shows statistically obvious differences between two biological states (38). To further understand the underlying mechanisms of the 9 key mRNAs, such as CYP17A1 and TFPI2, in the ceRNA network, GSEA was performed. Firstly, we divided samples from the GSE56166 dataset into two groups based on the median expression level of these key mRNAs. Then, according to the normalized enrichment score (NES), false discovery rate (FDR) q-value, and nominal (NOM) P value, the five most significantly enriched signaling pathways associated with key genes, respectively, were identified (**Figure 10**).

GSEA results suggested that ASNS and CYP17A1 might participate in “complement and coagulation cascades”, “drug metabolism-cytochrome P450”, “drug metabolism-other enzymes”, “metabolism of xenobiotics by cytochrome P450”, and “PPAR signaling pathway”. DAPK1 might be related with “complement and coagulation cascades”, “drug metabolism-cytochrome P450”, “olfactory transduction”, and “PPAR signaling pathway”. HBB might participate in “neuroactive ligand-receptor interaction” and “olfactory transduction”. HGF might only participate in “neuroactive ligand-receptor interaction”. PRL might be related with “gap junction”, “neuroactive ligand-receptor interaction”, “olfactory transduction”, and “systemic lupus erythematosus”. TFPI2 also might related with “complement and coagulation cascades”, “drug metabolism-cytochrome P450”, “neuroactive ligand-receptor interaction”, “olfactory transduction”, “PPAR signaling pathway”. VWF might associate with “complement and coagulation cascades”, “drug metabolism-other enzymes”, “neuroactive ligand-receptor interaction”, “PPAR signaling pathway”, and “prion disease”. TNFAIP6 might be involved in “allograft rejection”, “cytokine-cytokine receptor interaction”, “drug metabolism-other enzymes”, “primary immunodeficiency”, and “T cell receptor signaling pathway”.

## Discussion

Osteoporosis is one of the most common systemic metabolic bone diseases (39), especially in postmenopausal women (40). Osteoporosis occurs due to excessive bone resorption and impaired bone formation (41). Accumulating evidences have suggested that dysregulated circRNAs are associated with the occurrence and progression of osteoporosis (42, 43). However, the specific role of the circRNA-related network remains mostly undescribed in osteoporosis. Furthermore, various studies nowadays are assessing the circRNA-miRNA or TF-mRNA regulatory networks, but fewer studies focus on the miRNA-TF co-regulatory network. Herein, we constructed a circRNA-miRNA-TF-mRNA regulatory network using bioinformatics analysis approaches. The network may help to elucidate the key roles of circRNAs in the pathogenesis of PMOP and may help to guide the diagnosis and treatment of PMOP.

We first downloaded PMOP-related datasets, GSE161361, GSE64433, and GSE56116, from the GEO database, which were used for analysis to obtain differentially expressed circRNAs, miRNAs, and mRNAs. A total of 1201 differentially expressed mRNAs were identified in PMOP patients compared with the control group, including 935 upregulated and 266 downregulated mRNAs. We applied GO enrichment analysis on selected differentially expressed mRNAs, the results showed that enriched mRNAs were involved in many biological processes, which included “positive regulation of cytokine production”, “active regulation of IL-6 production”, and “regulation of chemotaxis”, “regulation of peptidase activity”. Besides, the most common molecular functions for these DE mRNAs included “Toll-like receptor binding”, “endopeptidase regulator activity”, “endopeptidase inhibitor activity”, and “chemoattractant activity”. Most of the biological processes and the main molecular functions are related to inflammatory responses. Previous studies also suggested that inflammation plays a crucial role in the progression of OP. Human and animal experiments have shown pro-inflammatory cytokines are crucial mediators of the accelerated bone loss in PMOP, such as interleukin-6 (44). Some proinflammatory cytokines, pathogen-associated molecular patterns, or endogenous pathogenic factors can induce osteoporosis by binding to the Toll-like receptors (TLRs) (45).

The results of the KEGG pathway enrichment analysis illustrated that DE mRNAs were mainly related to some pathways, such as “osteoclast differentiation”, “rheumatoid arthritis”, “hematopoietic cell lineage”, and “cytokine-cytokine receptor interaction”. Previous studies have also supported the roles of these pathways in osteoporosis. Hyper-differentiation of osteoclasts is the hallmark of PMOP (46). Osteoclasts are specialized cells derived from hematopoietic cell lineage, specifically from the monocyte/macrophage lineage, which adhere to the bone matrix and resorb the bone (47). Genes enriched in the hematopoietic cell lineage pathway, such as the CSF-1 receptor (CSF-1R) can regulate the development of osteoclasts (48). In addition, osteoporosis is generally common in patients with rheumatoid arthritis which is a systemic inflammatory disease, and inflammation is also mediated by the Toll-like receptor signal (49). The other significant pathway was cytokine-cytokine receptor interaction, which is involved in inflammatory host defenses, cell growth, differentiation, and angiogenesis aimed at restoration of homeostasis (50). Some studies have confirmed genes enriched in this pathway contribute to the development of osteoporosis (51).

Transcription factors (TFs) are proteins that can bind to gene-specific sequences, also known as promoters of genes, and thus mediate genes transcription and expression (52). Numerous studies have demonstrated that several transcription factors play a regulatory role in the pathogenesis of OP (53). Hence, to further identify key transcription factors in PMOP, we obtained human TF-mRNA pairs among differentially expressed mRNAs by TRRUST v2 database, which is now believed to be the most comprehensive database based on manual curation for TF-target interactions in humans and mice (54). Here we first identified 26 transcription factors and their target mRNAs. MiRNAs could target TF and form miRNA-TF gene expression regulatory circuits to regulate target gene expression. Combining DE miRNAs, we next obtained miRNA-TF/mRNA pairs, which include 9 miRNAs and 12TF/mRNAs. Combining DE circRNAs, we constructed the ceRNA network containing 6 circRNAs, 4 miRNAs, 4 TFs, and 12 mRNAs.

CTD is a powerful public database that aims to improve understanding of how environmental exposure affects human health (55). The results of CTD further suggested that these 4 TFs and 12 mRNAs are associated with postmenopausal osteoporosis. Furthermore, we verified the expression of candidate genes in the network in PMOP and control samples using qRT-PCR. Except for hsa_circ_ 0039035, hsa_circ_ 0086166, HMOX1, SPI1, and SLC19A1, the expression levels of other circRNAs, miRNAs, mRNAs, and TFs were consistent with the microarray results. Combined with the PCR validation results, the ceRNA network was finally constructed containing 4 circRNAs, 3 miRNAs, 3 TFs, and 9 mRNAs. The core mRNAs in the ceRNA network may contribute to osteoporosis. For example, hepatocyte growth factor (HGF) has been highlighted to relieve bone loss in the early stages of PMOP mouse models (56). Previous studies have reported that prolactin (PRL) is related to osteoporosis risk (57). TNF alpha induced protein 6 (TNFAIP6) is implicated in bone loss and the pathology of osteoporosis in aging (58). As a target gene of lncRNA SNHG5, hemoglobin subunit beta (HBB) may be involved in the development of male osteoporosis (59). Cytochromes P450 (CYPs) are important for bone homeostasis (60), and cytochrome P450 family 17 subfamily A member 1(CYP17A1) is thought to be a susceptibility loci for altered BMD in PMOP patients (61). In previous studies, there was no direct evidence for the role of other genes, such as VWF, TFPI2, ASNS, and DAPK1 in PMOP.

Next, to further understand the underlying mechanisms of the 9 key mRNAs in the ceRNA network, GSEA was performed. The Gene set enrichment analysis (GSEA) revealed that these differentially expressed genes participate in many important signaling pathways. Previous studies have shown that the occurrence and development of PMOP are related to “olfactory transduction” (62), “systemic lupus erythematosus” (63), and “T cell receptor signaling pathway” (64). Besides, the pathway of neuroactive ligand-receptor interaction may involve in osteoclastogenesis (65). In GC-induced osteoporosis, PPAR-γ could affect osteogenic differentiation (66). Gap junctions play a significant role in bone development and function, research showed that gap junction modulation may be a promising new target for osteoporosis therapy (67). In summary, key mRNAs in the ceRNA network may participate in the development of osteoporosis by regulating related signal pathways.

The core transcription factors in the ceRNA network, NFIC, BACH1, CEBPB, and POU2F2, might be related to osteoporosis. NFIC (nuclear factor I C) is a member of the nuclear factor I family of transcription proteins (68). NFI-C serves as one of the vital transcription factors for postnatal bone formation and bone homeostasis (69). A previous study found the expression of NFIC was decreased in osteogenic cells from human osteoporotic patients (70). The deficiency of NFIC inhibited osteoblast differentiation and bone formation *in vivo* (70). CEBPB, also called CEBPβ (CCAAT/enhancer-binding protein β), belongs to the transcription factors family of the CCAAT/enhancer-binding proteins (71). Previous studies have proven that the C/EBPB is involved in lytic bone diseases, especially osteoporosis (72). C/EBPB can promote osteoblast differentiation (72). C/EBPB is also known as a switch in osteoclast differentiation (72). Moreover, as an adipogenic transcription factor, C/EBPB also can participate in adipogenic differentiation of bone marrow mesenchymal stem cells (73). POU2F2 (POU-2 Homeobox 2) belongs to the POU transcription factor family (74). There is no direct evidence that POU2F2 is involved in osteoporosis. Recent studies have shown that POU2F2 could promote glioblastoma progression by regulating glycolysis (74). The pathogenesis of osteoporosis is related to the dysregulation of glycolysis (75). Furthermore, POU2F2 expression is related to fracture healing, and overexpression of POU2F2 promoted protein and mRNA expression of Colla1, Runx2, Osterix, and Osteocalcin (76) in osteoporosis. These genes are associated with bone formation and osteoblastic differentiation. Combined with these previous studies, our results demonstrated correlations between these core TFs and PMOP. Further work will focus on the exact contribution of NFIC, BACH1, CEBPB, and POU2F2 to osteoporosis pathogenesis.

MiRNAs are key components in forming a ceRNA regulatory network (77). Undoubtedly, identifying the relationship between miRNAs and diseases can not only improve the understanding of molecular mechanisms and disease pathogenesis, but also be beneficial to clinical diagnosis and treatment (78). In our study, 4 miRNAs were finally identified in this circRNA-miRNA-TF-mRNA regulatory network, and they were hsa-mir-566, hsa-mir-4768-3p, hsa-mir-629-3p, and hsa-mir-623. No direct studies have addressed the roles of the 4 miRNAs in osteoporosis to date. Therefore, in our study, the association between miRNAs and PMOP was further validated by qRT-PCR experiments. Our results corroborated the involvement of these miRNAs in PMOP and shed new light on the role of these miRNAs in PMOP pathogenesis. However, how these miRNAs are involved in PMOP requires further carrying out experiments. In addition, as a complement to the experimental approaches, computational methods, such as the unsupervised deep learning model of the variational autoencoder for MiRNA–disease association prediction (VAEMDA) (78), and the computational model of random walk with restart for MiRNA–disease association (RWRMDA) (79) may also provide more help in probing PMOP-related candidate miRNAs in the following study.

Finally, we identified 4 circRNAs in the ceRNA network associated with the above miRNAs, which were hsa_circ_0023417, hsa_circ_0078309, hsa_circ_0063533, and hsa_circ_0036760. CircRNA is believed to be a new and promising hotspot in the field of non-coding RNA research, compared with known miRNA and LncRNA (80). Some circRNAs have been identified, but their specific regulatory roles are often poorly understood (81). Thefour circRNAs of the ceRNAnetwork identified in our research have not been presently reported to be associated with PMOP. Estrogen receptor 1(ESR1) is the host gene of hsa_circ_0078309, which is an estrogen receptor subtype widely expressed in bone tissue (82). Studies have illustrated that estrogen plays a role in regulating bone metabolism mainly by interacting with ESR1 (83). So, has_circRNA_0078309 may regulate bone metabolism of PMOP. Currently, no direct studies are reporting the role of hsa_circ_0023417, hsa_circ_0063533, and hsa_circ_0036760 in osteoporosis. Our data further support the possibility that the four circRNAs maybe important regulators of osteoporosis development. However, the precise mechanism of these circRNAs and their related network requires further study.To sum up, we constructed the ceRNA network jointed by 4 circRNAs, 3 miRNAs, 3 TFs, and 9 mRNAs. To our knowledge, this is the first time that a ceRNA network containing TFs has been established in osteoporosis., Combined with experimental validation and bioinformatics analysis, the findings suggest that the circRNA-miRNA-TF-mRNA regulatory network may be involved in PMOP and may be potential therapeutic targets of PMOP.

## Data Availability Statement

The datasets presented in this study can be found in online repositories. The names of the repository/repositories and accession number(s) can be found in the article/**Supplementary Material**.

## Ethics Statement

The studies involving human participants were reviewed and approved by Ethical Committee of Gansu Provincial Hospital. The patients/participants provided their written informed consent to participate in this study.

## Author Contributions

(I) Conception and design: QD, LT; (II) Administrative support: LT; (III) Collection and assembly of data: QD, ZH; (IV) Data analysis and interpretation: QD, ZH; (V) Manuscript writing: All authors; (VI) Final approval of manuscript: All authors.

## Fundings

This work was sponsored by the National Natural Science Foundation of China (No. 82060152), Clinical Research Center for Metabolic Diseases (18JR2FA006), and Lanzhou Talents Innovation and Entrepreneurship Project (2018-RC-79).

## Conflict of Interest

The authors declare that the research was conducted in the absence of any commercial or financial relationships that could be construed as a potential conflict of interest.

## Publisher’s Note

All claims expressed in this article are solely those of the authors and do not necessarily represent those of their affiliated organizations, or those of the publisher, the editors and the reviewers. Any product that may be evaluated in this article, or claim that may be made by its manufacturer, is not guaranteed or endorsed by the publisher.
