# Supplementary material for: Identification of Serum Exosome-Derived circRNA-miRNA-TF-mRNA Regulatory Network in Postmenopausal Osteoporosis Using Bioinformatics Analysis and Validation in Peripheral Blood-Derived Mononuclear Cells
Source: Front Endocrinol (Lausanne). 2022 Jun 9;13:899503. doi: 10.3389/fendo.2022.899503 (PMC9218277; doi:10.3389/fendo.2022.899503)
Supplement: Supplementary file 1 [file DataSheet_1.docx]

Supplementary data

S1. Genetic Dataset Details

| Series | Title | Organism | Platforms | Samples | Status |
| --- | --- | --- | --- | --- | --- |
| GSE56116 | Genes expression profiles of postmenopausal osteoporosis with kidney Yin deficiency | Homo sapiens | GPL4133 | GSM1356155  GSM1356156  GSM1356157  GSM1356158  GSM1356159  GSM1356160  GSM1356161  GSM1356162  GSM1356163  GSM1356164  GSM1356165  GSM1356166  GSM1356167 | Public on Mar 23, 2014 |
| GSE161361 | Identification of exosomal circRNAs as potential biomarkers for postmenopausal osteoporosis | Homo sapiens | GPL28148 | GSM4905321  GSM4905322  GSM4905323  GSM4905324  GSM4905325  GSM4905326 | Public on Nov 13, 2020 |
| GSE64433 | Detection of potential microRNA biomarker for postmenopausal osteoporosis | Homo sapiens | GPL18402 | GSM1571226  GSM1571227  GSM1571228  GSM1571229  GSM1571230  GSM1571231 | Public on Dec 23, 2014 |

S2. Characteristics of clinical samples

| Traits | PMOP group (n=6) | NC group (n=6) |
| --- | --- | --- |
| Age(years) | 61.37±6.37 | 55.03±5.28 |
| Height(cm) | 157.43±5.03 | 161.33±7.55 |
| Weight(kg) | 58.57±9.68 | 62.67±8.85 |
| Lumbar spine (L1-L4) T-score | -3.37±0.57 | -0.40±0.24 |
| Hip T-score | -2.34±0.54 | 0.18±0.71 |
| Femoral neck T-score | -2.10±0.37 | 0.52±0.89 |
